# Obesity as a predictive factor for chronic kidney disease in adults: systematic review and meta-analysis

**DOI:** 10.1590/1414-431X202010022

**Published:** 2021-02-26

**Authors:** K.R.D. Pinto, C.M. Feckinghaus, V.N. Hirakata

**Affiliations:** 1 Complexo Hospital de Clínicas da Universidade Federal do Paraná, Universidade Federal do Paraná, Curitiba, PR, Brasil; 2 Hospital de Clínicas de Porto Alegre, Porto Alegre, RS, Brasil

**Keywords:** Obesity, End-stage renal disease, Albuminuria

## Abstract

Chronic kidney disease (CKD) is one of the main chronic diseases affecting the world population due to its high prevalence and increasing morbidity. Similarly, obesity gained the interest of the scientific community as it directly or indirectly increases mortality from cardiovascular causes, and its prevalence characterizes a pandemic. The objective of this study was to investigate obesity measured by body mass index as a predictor for end-stage renal disease in the general adult population. A systematic review and meta-analysis was carried out by searching 10 databases for prospective or retrospective cohort studies, with no restrictions on the language of publication, including adults with obesity without previous renal disease and who evolved to CKD (diagnosed by estimated glomerular filtration rate below 60 mL&mac_middot;min^-1^&mac_middot;(1.73 m^2^)^-1^ over the follow-up period. The R software and Meta package were used for data analysis. After removing duplicates, 5431 studies were submitted to the steps of the systematic review, and 21 articles were included in the data analysis. In total, 3,504,303 patients, 521,216 with obesity, and an average follow-up time of 9.86 years were included. The relative risk of obese people for developing CKD in the random effects model was 1.81 (95%CI: 1.52-2.16). The evidence found in this meta-analysis confirmed that obese people are at higher risk of developing CKD that the non-obese population (1.81 times higher), with obesity being a priority risk factor in preventive actions.

## Introduction

### Chronic kidney disease

Chronic kidney disease (CKD) is one of the main chronic diseases that affects the world population. In the United States, according to the National Health and Nutrition Examination Survey, new cases of CKD doubled in individuals over 65 years old from 2000 to 2008 and the prevalence of CKD in the population over 60 years of age went from 18.8% in 2003 to 24.5% in 2006.

Based on the 2018 census of the Brazilian Nephrology Society, 133,464 patients were estimated to be under treatment in 781 dialysis units in Brazil, with 80% of dialysis treatments being funded by the Public Health System (1).

### Obesity

The most traditional measure for obesity diagnosis is body weight adjusted for height - the body mass index (BMI) - calculated as: weight in kilograms divided by the square of the height in meters (2). According to the World Health Organization, body type is classified based on BMI in 5 categories: <18.5 kg/m^2^ underweight, 18.5 to 24.9 kg/m^2^ normal weight, 25 to 29.9 kg/m^2^ overweight, and >30 kg/m^2^ obesity (3). In Asian countries, a different classification is adopted using different cut-offs (4).

Different mechanisms are involved in the relationship between obesity and CKD. Indirectly, obesity can mediate the onset and worsening of diabetes mellitus and systemic arterial hypertension, and directly, it can lead to structural and inflammatory changes (5).

The obesity and CKD relationship can appear as a paradox: in the early stages of renal dysfunction, obesity contributes to glomerulopathy while in end-stage renal disease, obesity acts as a protective factor that increases survival. Possible causes of the protective effect of obesity in advanced CKD include better hemodynamic stability, alteration of circulatory cytokines, protein-energy wasting and inflammation, and time discrepancy among competitive risk factors (undernutrition *vs* overnutrition), optimizing clinical performance (6).

Obesity-related renal injury may present a circulatory component (vessel injury or compression by adipose tissue) (7), an inflammatory component (activation of inflammatory cytokines) (8), and a hormonal component (effect on the renin-angiotensin system) (9).

## Material and Methods

### Eligibility

Prospective or retrospective observational studies assessing the association between obesity and CKD were included without limitations on the publication date. The target population was obese adults with normal renal function at the beginning of the follow-up, with a minimum follow-up of 3 years, who progressed to CKD stages 3 to 5 at the end of the research. Studies that included patients undergoing renal replacement therapy, that evaluated bariatric surgery as obesity treatment in parallel with CKD, and that included patients with kidney transplant as past medical history at baseline were not included in the meta-analysis. The studies that included patients who underwent bariatric surgery during the study were excluded from the final analysis.

### Predictors and outcome

#### Predictor or exposure factor

Obesity, defined as a BMI above 30 or above 27.5 (for Asian studies), was the study predictor. The classification adopted in this study was in accordance with that recommended by the World Health Organization (3) but included an additional subgroup of BMI from 35 to 39.9, as obesity management for this subgroup differs from the other subtypes.

In the search parameters, all individuals with grade I, II, or III obesity were included under the obese group. Studies with a diagnosis of obesity based on regional BMI limits were also included, especially those from Asian countries.

#### Outcome

Glomerular filtration rate (GFR) is a measure of the flow rate of the fluid filtered by the glomeruli that is not reabsorbed or secreted while in the tubules. GFR is considered the gold standard for assessing renal function (10). The estimated GFR can be calculated by the Cockroft-Gault, MDRD, or CKD-EPI formulas, all of which establish the CKD classification and stage (11). In southern Brazil, the CKD-EPI formula is more accurate in estimating GFR compared to the MDRD equation (12). The primary outcome of this meta-analysis was the diagnosis of stages 3 to 5 CKD (11; Table 1), which included estimated GFR at least below 60 mL․min^-1^․(1.73 m^2^)^-1^ or need for renal replacement therapy (dialysis or renal transplantation).

**Table 1:** Chronic kidney disease (CKD) stages according to glomerular filtration rate.

Stage	Glomerular filtration rate [mL․min^-1^․(1.73 m^2^)^-1^]	CKD stage
1	>90	Normal renal function
2	60-89	Mild CKD
3	30-59	Moderate CKD
4	15-29	Severe CKD
5	<15	End-stage renal disease or dialytic CKD

Adapted from Romão Junior, 2004 (11).

### PROSPERO registration and ethics approval

This review was registered in the PROSPERO database with the code PROSPERO 2018 CRD42018091865; the study protocol and methodology can be accessed at http://www.crd.york.ac.uk/PROSPERO/display_record.php?ID=CRD42018091865. The study received ethics approval with the code CAAE 81020017.2.0000.5327 and title “Obesity as a predictor of chronic kidney disease: systematic review and meta-analysis” and was approved by the Research Ethics Committee of the Hospital de Clínicas de Porto Alegre (No. 2,455,676).

### Search strategies

The search terms used in the study prioritized sensitivity over specificity, with the aim to include any and all available evidence.

#### Databases and sources

A search was conducted in 10 databases that contained studies in the health field: PubMed Medline, Virtual Health Library (VHL), LILACS, Web of Science, Google Academics, Scopus, The Cochrane Library, Ovid, Scielo, and ProQuest. Hard data sources, such as bibliographic references of studies and the Thesis Bank of the São Paulo University (http://www.teses.usp.br) were also searched.

#### DeCS and MESH terms

The following MeSH or DeCS terms were used in Portuguese, English, or Spanish and according to strategies detailed in Supplementary Table S1: Albuminuria, Albuminúria, Body Mass Index (BMI), Chronic Kidney Disease, Chronic Kidney Failure, Chronic Kidney Insufficiency, Chronic Renal Insufficiency, Falência Renal Crônica, Fallo Renal Crónico, ĺndice de Masa Corporal, ĺndice de Massa Corporal, Insuficiencia Renal Crónica, Insuficiência Renal Crônica, Obesidade, Obesidad, Obesity, Overweight, Quetelet’s Index, and Sobrepeso.

The terms Quetelet’s Index, Chronic Kidney Disease, CKD, Chronic Kidney Insufficiency, End-Stage Kidney Disease, ESRD, Chronic Renal Disease, Chronic Renal Failure, Chronic Renal Insufficiency, Renal Impairment, and Kidney Injury were also searched as descriptors but were not registered as keywords or were used as synonyms in the databases.

#### Search fields and filters

The terms were searched for in the title, summary, and keywords with no filters for language and study dates, making it possible to include articles published in any past time.

#### “OR” and “AND” Boolean operators

The factors related to exposure and those related to the outcome were connected by “OR”, generating two search phrases - exposure and outcome. The phrases originated were then connected to each other with “AND”, maintaining the objective of establishing a causal relationship.

#### Search strategies

The search strategies for each database are shown in Supplementary Table S1.

### Paired review and quality assessment

A paired review was conducted by the researchers K.R.D. Pinto and C.M. Ferkinghaus. The studies were classified according to the quality parameters of the AMSTAR scale (13) in the following steps: i) Review of title and abstract; ii) Full-text reading of selected papers to verify the design, methodology, and outcome; iii) Quality assessment of the studies included for the systematic review and meta-analysis using the Newcastle-Ottawa scale. Divergences between researchers were discussed to reach a consensus.

### Methodology quality assessment by the AMSTAR scale

The AMSTAR scale (13) assesses 11 items of the methodology, is widely used in study quality assessment, and has been shown to have external validity. The results of the AMSTAR scale for our systematic review are shown in Supplementary Table S2.

### Screening of the articles by the title and abstract

Articles that had statements about which the researchers disagreed were classified as potentially eligible and selected for full-text reading.

### Eligibility and inclusion by reading the full text

After excluding experimental studies, editorials, and review articles, studies were assessed for design and eligibility criteria. Finally, articles were included for quality analysis and data extraction.

### Quality assessment

The included articles were submitted to quality assessment using the Newcastle-Ottawa scale (NOS). This scale was developed by the collaboration between the Universities of Newcastle (Australia) and Ottawa (Canada) to analyze the quality of non-randomized studies. A “star system” judges three perspectives: the selection of the study groups, the comparability of the groups, and the ascertainment of either the exposure or outcome of interest for the study, respectively (14).

### Data extraction

The data about year, country and continent of publication, design, age at baseline, age group, length of follow-up, obesity criterion, sample size, BMI classification, number of individuals who progressed to CKD, and quality classification were extracted from the selected studies.

### Statistical analysis

A meta-analysis of risk ratio (RR) for the development of CKD was carried out. The I^2^ was calculated for heterogeneity verification. Due to heterogeneity, regressions were performed with the following variables: 1) years of follow-up; 2) baseline mean age; 3) cohort design; 4) quality score (NOS); 5) continent of study publication; 6) obesity criteria; and 7) GFR formula used. Subgroup analyses were done to interpret the heterogeneity. The Meta command of the R software (v3.6.1; R Foundation, 2019; <https://www.r-project.org/foundation>) and the Meta package (v4.13-0; Schwarzer, 2020, <https://www.rdocumentation.org/packages/meta/versions/4.14-0>) were used for data analysis and the significance level was 0.05.

### Publication bias analysis

The publication bias analysis is presented in a Funnel plot in Supplementary Figure S1.

## Results

The search in the 10 databases retrieved 12,215 articles. The number of articles returned in each database is described in Supplementary Table S1.

**Figure 1:**
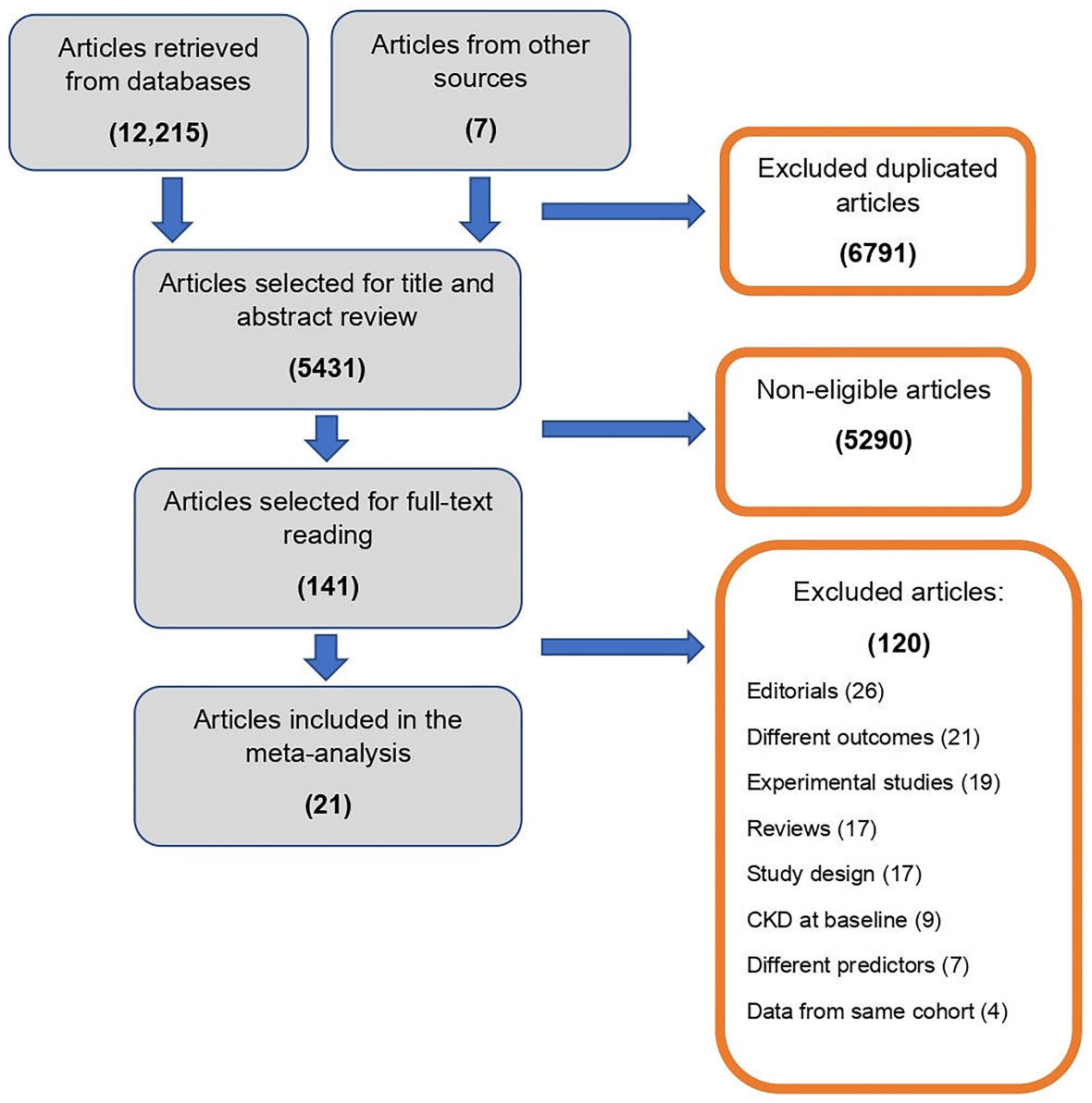
Flowchart of study selection by systematic review. CKD: chronic kidney disease.

After removing duplicates, 5,431 studies were screened by titles and abstracts and 141 studies underwent full-text evaluation. The Kappa coefficient (15) for the agreement of the results of title and abstract screening by the reviewers was 0.77. Seventy-nine studies were assessed for design, controls, and follow-up time, after which 21 articles were eligible for the meta-analysis. The reasons for article exclusion are shown in Figure 1.

### Study characteristics

Supplementary Table S3 shows the characteristics of the 21 studies included in the meta-analysis (16–36).

### Forest plot analysis

**Figure 2:**
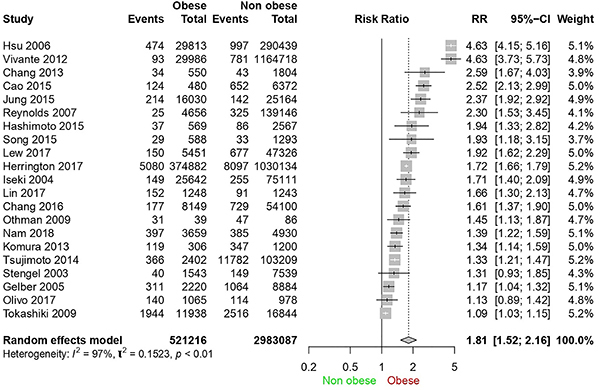
Forest plot of chronic kidney disease cases in obese and non-obese patients.

Figure 2 shows the forest plot of all study participants (3,504,303 individuals) divided into “obese” and “non-obese” groups, as well as the “events” subgroups, with the participants who developed CKD within each group at the end of the follow-up period. Based on the graph and the Q test (P<0.01), the null hypothesis of homogeneity (P>0.05) was rejected. A meta-analysis was carried out with a random effects model due to heterogeneity (I^2^=97%).

In two studies (29 [Bibr B30] [Bibr B31] [Bibr B32],^33^ [Bibr B34]), no statistical difference was found regarding exposure to obesity and CKD. In the other articles, the relative risk ranged from 4.63 to 1.09. The overall risk ratio of 1.81 (95%CI: 1.52-2.16) indicates that the probability of developing CKD of an obese person is 1.81 times higher than that of a non-obese person.

### Heterogeneity and meta-regression

Considering the 97% heterogeneity, the sensitivity assessment was done by subgroup analyses. The mean age at baseline was the variable with the strongest relationship with heterogeneity, being responsible for explaining 47.19% (r^2^=0.4719) of the variation.

Years of follow-up, mean age at baseline, and study design significantly influenced relative risk (Table 2) and were thus used for sensitivity analyses. The meta-regression with the GFR formula, years of follow-up, and year the study was carried out did not show any influence on the final heterogeneity; only a publication year later than 2015 had a slight effect on heterogeneity (Table 3).

**Table 2:** Meta-regression results of the obesity-chronic kidney disease relationship.

	QM	P	Estimated (95%CI)
Years of follow-up	27.7489	<0.0001	(subgroups in Table 3)
Publication year	1.6030	0.4487	(subgroups in Table 3)
Year study was carried out	1.4159	0.4927	(subgroups in Table 3)
Mean age at baseline	16.3828	<0.0001	-0.0273 (-0.0405;-0.0141)
Study design	8.2886	0.0040	0.4757 (0.1518;0.7995)
Newcastle-Ottawa scale	0.0228	0.8799	0.0175 (-0.2099;0.2450)
Continent	4.7871	0.1881	
Asia			-0.0687 (-0.5359;0.3985)
Europe			-0.1441 (-0.8745;0.5864)
Middle East			0.9244 (-0.0369;1.8857)
Obesity criterion	2.2060	0.1375	0.0521 (-0.0167;0.1209)

QM: quality management.

**Table 3:** Meta-regression results for subgroups (random effects model).

	K	RR	95%CI	Q	Tau^2^	I^2^
Formulas used for GFR estimation						
CKD-EPI	6	1.6807	(1.4116; 2.0010)	34.44	0.0352	85.5%
MDRD	12	1.7008	(1.2604; 2.2949)	622.13	0.2660	98.2%
Dialysis	3	2.6378	(1.2909; 5.3902)	47.14	0.3775	95.8%
Years of follow-up						
<9	9	1.7168	(1.4723; 2.0019)	70.08	0;0410	88.6%
9-14	8	1.4013	(1.2129; 1.6190)	53.25	0.0332	86.9%
>14	4	3.2434	(1.9854; 5.2985)	78.22	0.2338	96.2%
Publication year						
<2010	7	1.7177	(1.0309; 2.8619)	568.50	0.4599	98.9%
2010-2015	8	2.1581	(1.5474; 3.0098)	148.70	0.2086	95.3%
>2015	6	1.5707	(1.3879; 1.7774)	23.70	0.0168	78.9%
Year study was carried out						
<2000	4	2.2284	(1.1194; 4.4361)	111.98	0.4723	97.3%
2000-2010	5	1.7509	(1.2051; 2.5439)	175.90	0.1707	97.7%
>2010	10	1.7888	(1.5703; 2.0378)	57.17	0.0313	84.3%

GFR: glomerular filtration rate.

**Figure 3:**
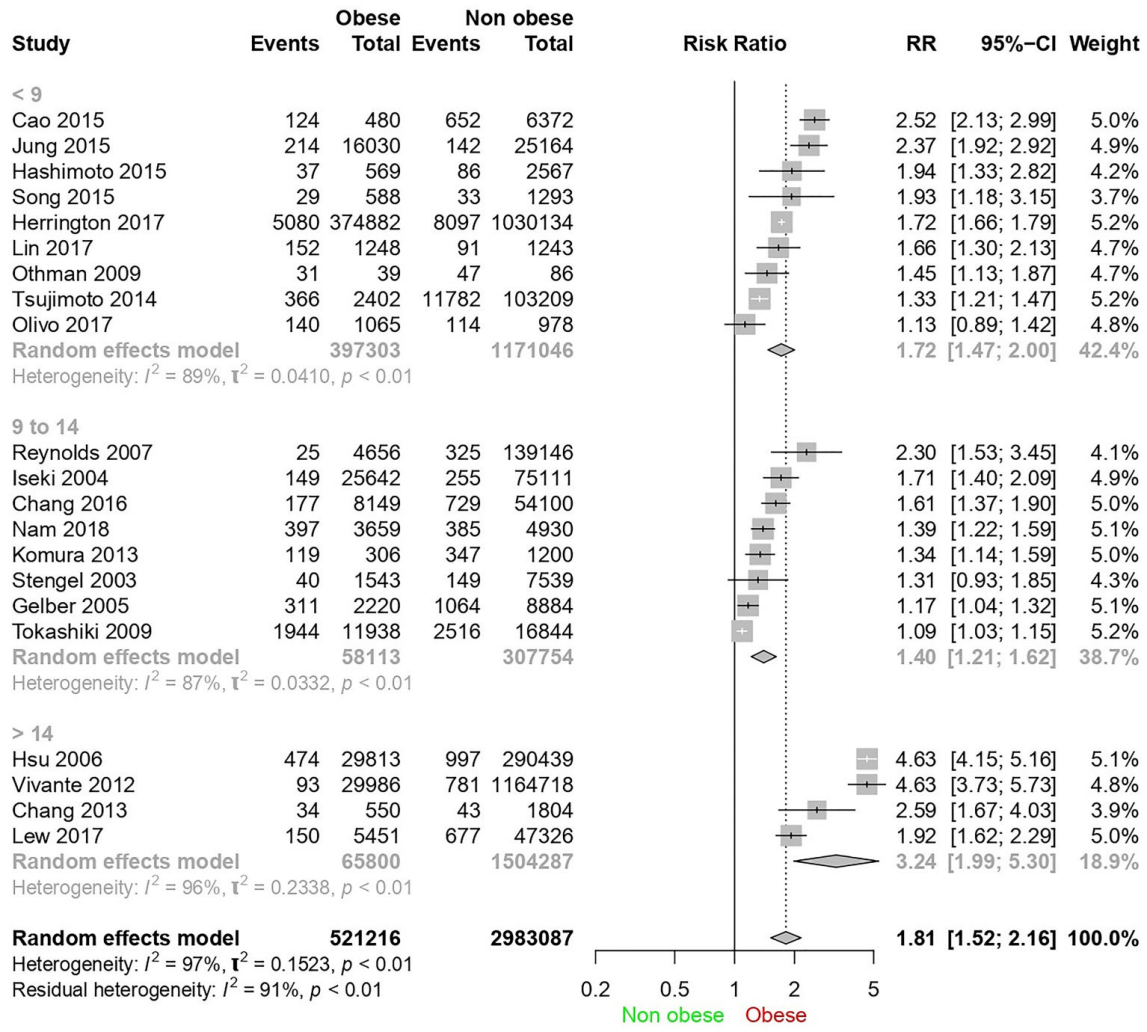
Forest plot for sensitivity analysis according to years of follow-up.

The first sensitivity assessment was done according to years of follow-up (Figure 3). Heterogeneity persisted at high levels (above 87% in all subgroups) and relative risk in the subgroup over 14 years of follow-up was 3.24 (95%CI: 1.99-5.30) while in the global analysis it was 1.81 (95%CI: 1.52-2.16).

**Figure 4:**
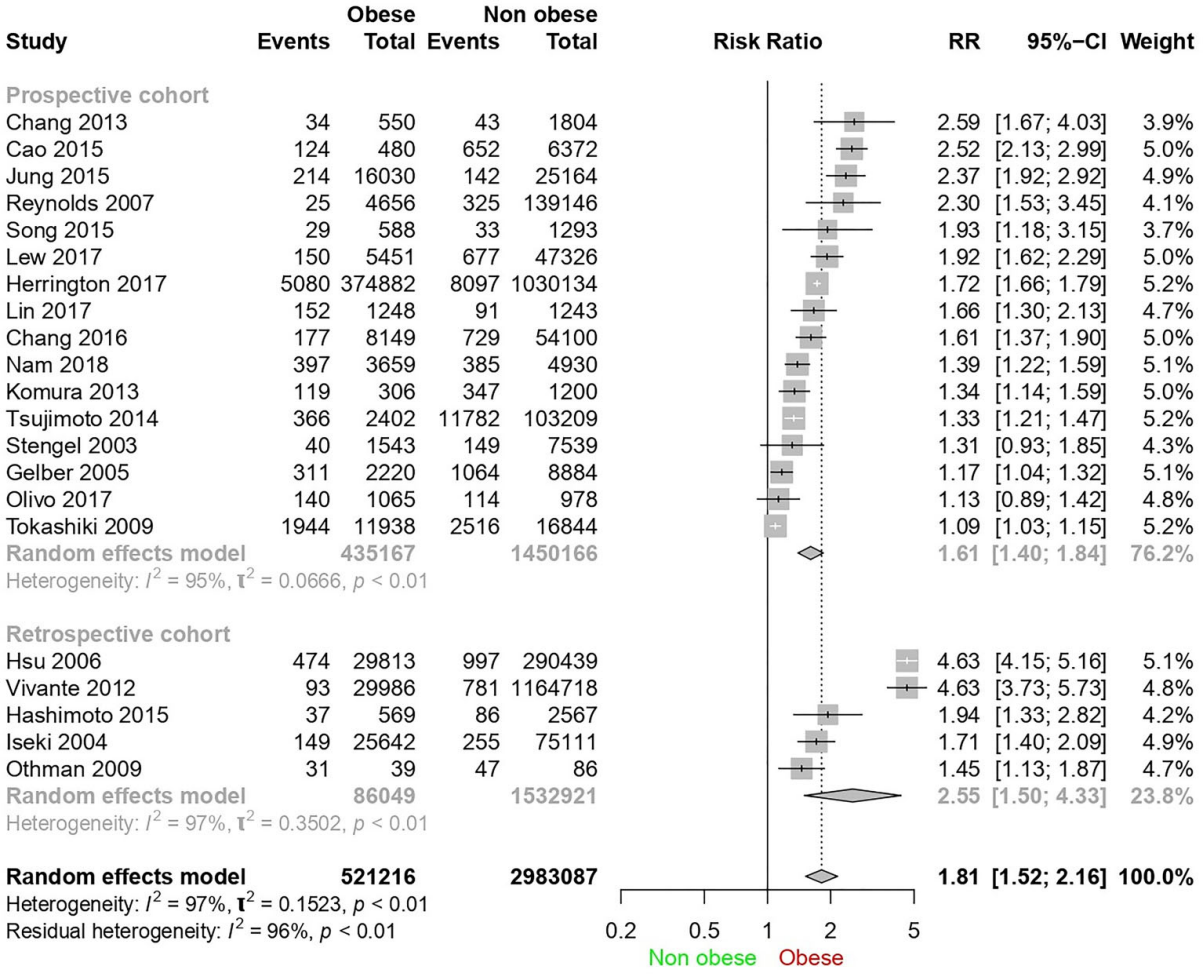
Forest plot for sensitivity analysis according to study design.

Another sensitivity assessment was related to study design (prospective and retrospective cohorts) (Figure 4). Heterogeneity was maintained for prospective cohorts (I^2^=95%) and for retrospective cohorts (I^2^=97%), showing that heterogeneity was not due to study designs. A higher relative risk was found in retrospective studies (2.55, 95%CI: 1.5-4.33) than in the global analysis.

**Figure 5:**
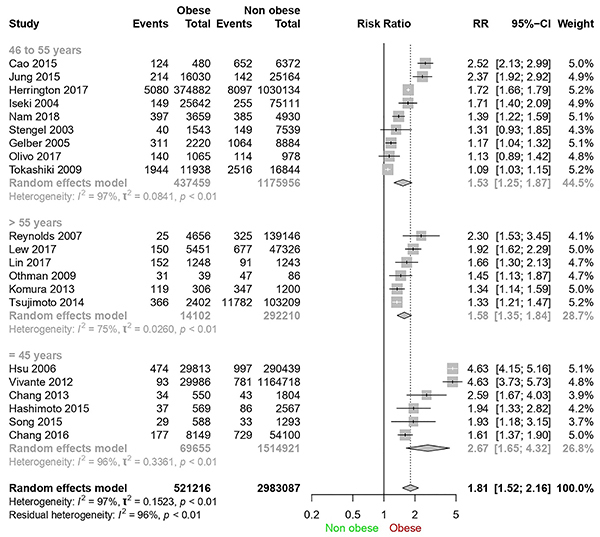
Forest plot for sensitivity analysis according to mean age range.

Analysis was carried out by age subgroups: i) up to 45 years; ii) from 46 to 55 years; and iii), above 55 years (Figure 5). An I^2^ of 75% was found for the over-55-year-old subgroup and it was the lowest heterogeneity index present when comparing the three age groups.

**Figure 6:**
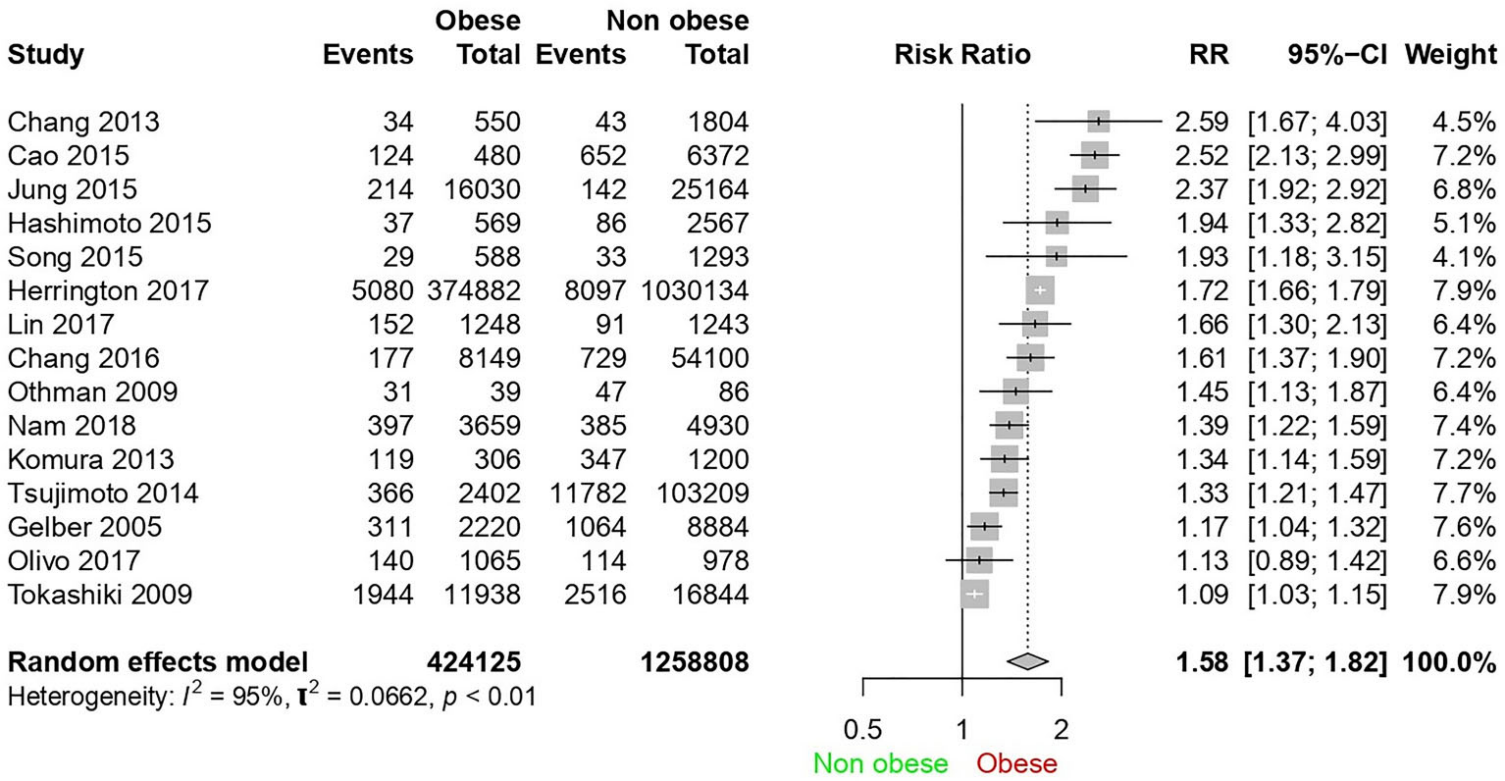
Forest plot for complementary sensitivity analysis excluding articles with kidney transplantation, dialysis, and estimated glomerular filtration rate below 15 mL&mac_middot;min^-1^&mac_middot;(1.73 m^2^)^-1^ outcomes.

Complementary sensitivity analysis (Figure 6) demonstrated persistence of high heterogeneity (95%) and relative risk of 1.58 even excluding articles with other outcomes such as kidney transplantation, dialysis, and eGFR below 15 mL․min^-1^․(1.73 m^2^)^-1^.

## Discussion

### Comparison of the results with previous studies

The 21 studies included in the meta-analysis were published between 2003 and 2018, showing that obesity as a risk factor for CKD is a relatively recent subject. Other systematic reviews and meta-analyzes also included recent studies (37,38).

This study also had differential points in comparison with other meta-analyzes: articles from Asia were included, the outcome of interest of the present study was CKD stages 3 to 5 and not necessarily associated with the criteria of albuminuria, studies in languages other than English were searched, and metabolic syndrome was not used as a synonym for obesity.

The relationship between obesity and CKD found in this study is in accordance with the existing literature (37).

In only two studies (29,33), no statistical difference was found regarding exposure to obesity and CKD. In one study (29), the research objective was the relationship between visceral obesity, not just BMI, in the development of CKD. The conclusion describes the increased risk to CKD in elevated BMI, but failed to establish a statistical correlation, probably limited by the sample size. In the other study (33), they found a correlation between morbid obesity and the risk for CKD, but risk was not increased for those classified as overweight or obese. However, the study had limited power in subgroup analysis, which could impact the interpretation.

The presence of several studies from Asia indicates the interest on this theme by that population. In addition, numerous studies from Asian countries also have cross-sectional designs for epidemiological purposes (39).

The studies included in meta-analysis articles were submitted to quality assessment (14) and were classified as having adequate methodological quality or higher. Finally, the finding that obesity confers an increased risk for CKD agrees with the previously published studies, even when accounting for metabolic state.

### Potential study limitations

Among the potential study limitations, our meta-analysis did not include studies carried out in Brazil or Latin America because none could be found through our search strategy; this limits the reproducibility of the results and medical recommendations for that population. In addition, the clinical heterogeneity of the included patients can be a source of inconsistency between the findings of observational studies.

On the other hand, we can list several limitations observed in individual studies. First, detailed information on use of medications was not collected in some studies because of the nature of community-based health research (35). Second, calibration of dipstick urinalysis and creatinine laboratory tests differed between studies and even in each study there may have been differences in techniques, despite the established parameters to consider normality (21). Third, we observed that many studies had patients with only one or two BMI measurements, however, within-person correlation of BMI over time is high (22 [Bibr B23] [Bibr B24] [Bibr B25] [Bibr B26] [Bibr B27] [Bibr B28]). Finally, the population restriction presented by some studies may limit their generalizability (20).

Such limitations can be overcome by selecting studies with similar methodologies in the systematic review (40), which was done in our study by including cohort studies with the healthy adult population and exposure to obesity. Nevertheless, factors such as sample size, average age of participants, or the continent of the study may have some influence on heterogeneity. However, further studies evaluating the correlation between obesity and CKD in Brazil or Latin America are required to reinforce these findings.

### Conclusions

This meta-analysis supports the hypothesis that obesity might be a risk factor to CKD and must be a priority in preventive actions. Public health measures targeting body weight control in the general population may contribute for preventing the increasing of CKD.

## Supplementary Material

Click here to view [pdf].
